# *Salmonella* pathogenicity Island 1 undergoes decay in serovars adapted to swine and poultry

**DOI:** 10.1128/spectrum.02643-24

**Published:** 2024-12-11

**Authors:** Martina Tambassi, Melissa Berni, Chiara Bracchi, Ilaria Menozzi, Alessandra Dodi, Laura Mazzera, Marina Morganti, Erika Scaltriti, Stefano Pongolini

**Affiliations:** 1 Risk Analysis and Genomic Epidemiology Unit, Istituto Zooprofilattico Sperimentale della Lombardia e dell'Emilia-Romagna (IZSLER), Parma, Italy; University of Guelph College of Biological Science, Guelph, Ontario, Canada

**Keywords:** *Salmonella enterica*, *Salmonella* Pathogenicity Island 1, SPI-1, food-borne pathogens, host-adaptation, *Salmonella* risk assessment, livestock

## Abstract

Human salmonellosis is a high-priority foodborne disease worldwide. The main reservoir of *Salmonella* is livestock, mainly swine and poultry that are infected both by generalist serovars and serovars adapted to them. The most widespread livestock-adapted serovars are attenuated in both their primary hosts and humans. We previously identified a lineage of the swine-adapted *Salmonella* Derby carrying mutations in *Salmonella* Pathogenicity Island-1(SPI-1) giving attenuation. To evaluate if SPI-1 decay is a general feature of swine-adapted serovars, we analyzed the Enterobase global population of *Salmonella* serovars most frequently isolated from swine. We found that deleterious mutations in SPI-1 are accumulated more in swine-adapted (*S*. Derby and *Salmonella* Rissen) than in generalist (*Salmonella* Typhimurium, I 1,4,[5],12:i:-, and *Salmonella* Infantis) serovars. The genomes carrying such mutations are widespread in the population of *S*. Derby and *S*. Rissen, indicating that the occurrence and fixation of deleterious mutations in SPI-1 are frequent. Similarly, also poultry-adapted *Salmonella* Kentucky has accumulated deleterious mutations in SPI-1, leading to over 70% of mutated genomes of this serovar compared with negligible proportions of mutated genomes in the generalist serovars from poultry. Although *in vitro* attenuation of *S*. Derby carrying mutated SPI-1 had been previously demonstrated, we showed the same phenotype for mutated *S*. Rissen. Considering also the known attenuation of *S*. Kentucky, our population-scale findings provide evidence of progressive evolution toward the attenuation of serovars adapted to the main animal reservoirs of human salmonellosis through the food chain. Intensive farming with a high density of animals could be a possible driver of this evolution.

**IMPORTANCE:**

This study shows at the global population level of *Salmonella* that the main attenuated serovars adapted to food-producing animals are undergoing convergent evolution toward further attenuation through the decay of SPI-1, considered critically important for the intestinal phase of *Salmonella* infection. The drivers of this evolution are unknown, but they could be attributed, at least in part, to the intensive farming of livestock with its high densities. On one side, our results contribute to the knowledge of the interaction between livestock populations and their host-adapted serovars of *Salmonella*. On the other side, the study provides scientific background for reconsidering the microbiological criteria adopted by the food safety legislation of many countries that ask for the absence of *Salmonella* in foods, regardless of any virulence evaluation of the detected strain. In this respect, the study provides molecular bases to investigate the virulence of different lineages within these host-adapted serovars.

## INTRODUCTION

*Salmonella enterica* subspecies *enterica* is an important pathogen for animals and a high-priority causative agent of foodborne disease in humans worldwide, ranking second for the number of cases in the European Union (EU) and the United States, according to recent data (1, 2). The main source of human salmonellosis is food of animal origin, mostly derived from poultry, swine, and bovine. Despite *S. enterica* including over 2,600 serovars, these animal species are mostly infected by a few, common serovars. These common serovars differ in their host spectrum, that is, the range of host species, including humans, in which they circulate and persist (3, 4). *Salmonella* Enteritidis, *Salmonella* Typhimurium, the monophasic variant of *S*. Typhimurium (I 1,4,[5],12:i:-) and *Salmonella* Infantis are generalist serovars because they are frequently isolated from more hosts. Conversely, *Salmonella* Derby and *Salmonella* Rissen are considered swine-adapted serovars as they are mainly found in swine (5, 6), whereas *Salmonella* Kentucky is a poultry-adapted serovar (7, 8). These differences in host adaptation appear to go along with important differences in the health impact of these serovars in humans, where the generalist *S*. Enteritidis, *S*. Typhimurium, I 1,4,[5],12:i:-, and *S*. Infantis caused 54.6%, 12.1%, 10.4%, and 2.3% of salmonelloses in EU in 2022, respectively, whereas the host-adapted *S*. Derby, *S*. Kentucky, and *S*. Rissen were responsible for only of 1.1%, 0.67%, and less than 0.35% of human cases, respectively (1). This evidence does not mean that these host-adapted serovars cannot infect humans but certainly confirms their reduced ability to cause disease. Moreover, *S*. Derby and *S*. Rissen are generally asymptomatic also in their main host, swine, similarly, *S*. Kentucky in poultry. Therefore, these serovars can be regarded as attenuated.

Virulence can vary significantly even within the serovar level. In previous studies, we detected substantial differences among genetic lineages of *S*. Derby belonging to sequence type 40 (ST40) (9, 10). In particular, we observed two lineages in Enterobase accounting for 24.9% and 5.3% of the entire *S*. Derby population from swine, which were underrepresented in humans compared with swine, indicating low pathogenicity for the human host. Those lineages show the accumulation of loss-of-function mutations in *Salmonella* Pathogenicity Island 1 (SPI-1) consistent with their attenuation. Specifically, they carry stop mutations or truncation/deletion of two SPI-1 genes. SPI-1 encodes the type three secretion system 1 (T3SS-1) and several effectors that *Salmonella* needs to invade and colonize the intestinal epithelium with high effectiveness to cause disease (11). We demonstrated that the observed mutations affect the ability of those lineages of *S*. Derby to infect intestinal epithelial cells *in vitro*. Therefore, we hypothesized that *S*. Derby could accumulate deleterious mutations in SPI-1 because this pathogenicity island could be not essential to circulate asymptomatically in pigs. In line with this hypothesis, it was previously demonstrated that SPI-1 plays a crucial role in the invasion and colonization of the porcine gut but not in the colonization of the tonsils where *Salmonella* is usually localized in healthy carrier pigs (12, 13). The accumulation of mutations in SPI-1 also contributes to attenuation for humans. Based on these previous observations, in this study, we further investigated the extent and characteristics of SPI-1 decay beyond *S*. Derby, including the main attenuated serovars adapted to swine, poultry, and bovine in comparison with the generalist serovars from the same species by analyzing the global population of *Salmonella* genomes available in Enterobase. In particular, the aim of the study was to evaluate if SPI-1 decay was restricted to a few genes in *S*. Derby or if it was a more extended and general process associated with many serovars adapted to different food-producing animal species and involving several genes.

## MATERIALS AND METHODS

### Analysis of SPI-1 in the global population of swine-, poultry-, and bovine-associated serovars

SPI-1 in the global population of serovars with the percentage of positive sampling units > 4% in swine (I 1,4,[5],12:i:-, *S*. Derby, *S*. Typhimurium, *S*. Infantis, and *S*. Rissen), poultry (*S*. Enteritidis, *Escherichia* Kentucky, *S*. Typhimurium, *S*. Infantis, *Salmonella* Mbandaka, and *Salmonella* Thompson), and bovine (*S*. Typhimurium, *Salmonella* Dublin, and *S*. Infantis), as reported by the last EU Zoonoses Reports, was analyzed by using the whole-genome sequences and tools available in the Enterobase database (https://enterobase.warwick.ac.uk, (14)), accessed between 6th and 24th May 2024. Specifically, genomes belonging to the serovars of interest were selected by using the serovar prediction performed by the Achtman 7 gene Multi Locus Sequence Type (MLST) scheme. Genomes with “swine,” “poultry,” or “bovine” as source type were selected, and for each genome, whole genome MLST (wgMLST) data were downloaded to analyze the alleles assigned to each of the 37 loci corresponding to SPI-1 genes. For *S*. Kentucky, genomes with “2021,” “2022,” and “2023” as collection years were analyzed. The remaining SPI-1 genes *stm2880*, *spaP*, and *invA* were not analyzed because they are not included in the wgMLST loci. Genomes carrying at least one truncated or missing allele of a SPI-1 gene were identified through the presence of the allele coded “−1” or “-,” respectively. Categorical data were recoded as numbers and percentages. Statistical analysis was performed using Fisher’s exact test in GraphPad PRISM 10.2.2.

The wgMLST trees of *S*. Derby, *S*. Rissen, and *S*. Kentucky were calculated in the Enterobase workspace using the GrapeTree option and selecting the Ninja neighbor-joining algorithm (15). For *S*. Kentucky, the phylogenetic analysis was conducted on a selection of 149 genomes randomly chosen from those previously selected for the statistical analysis.

### Bacterial strains

The bacterial strains used in this work belong to the collection of *Salmonella* isolates of human, animal, and environmental origin isolated in Emilia-Romagna Region from 2021 to 2024 as part of the official *Salmonella* surveillance conducted by our laboratory. Each strain of the collection is sequenced using an Illumina Nextseq platform (Illumina, San Diego, CA), and the reads are analyzed using Kraken for taxonomic classification (https://github.com/DerrickWood/kraken), followed by *in silico* typing through Achtman 7 gene MLST and core genome MLST (cgMLST) based on the Enterobase scheme. Three *S*. Rissen strains, one carrying wild-type SPI-1 (ER7016) and two naturally lacking the entire island (ER6778 and ER6482), were selected from our *Salmonella* collection for the phenotypic analysis. Strains were cultured in Luria Bertani (LB) Miller medium supplemented with ampicillin 100 µg/mL when needed.

### Mammalian cell cultures

INT-407 embryonic human intestine epithelial cell line and IPEC-J2 non-transformed swine intestinal epithelial cell line were purchased from the OIE collaborating Italian Biobank of Veterinary Resources. INT-407 cells were cultured in Minimum Essential Medium (MEM, Sigma-Aldrich) containing 10% fetal bovine serum, penicillin 100 U/mL, and streptomycin 100 µg/mL (pen/strep). IPEC-J2 cells were cultured in 50% Dulbecco’s Modified Eagle’s Medium (DMEM, high glucose, Sigma-Aldrich) and 50% Ham’s F12 Nutrient Mixture (Sigma-Aldrich) containing 5% fetal bovine serum, supplemented with pen/strep. Both cell lines were maintained at 37°C in 5% CO_2_ and used within 12 passages of receipt.

### Automated analysis of *Salmonella* intracellular phenotypes

The bacterial infection of INT-407 and IPEC-J2 cells for the quantification of *Salmonella* Intracellular phenotypes was performed according to Berni M et al. (16). Briefly, INT-407 and IPECJ-2 cells were seeded at 3 × 10^4^ and 1 × 10^4^ cells/well, respectively, in antibiotic-free media 1 day before the infection in 96-well imaging plates (CellVis) coated with collagen I from rat tail (Invitrogen). *Salmonella* strains to test for their intracellular phenotypes were transformed with the pCHAR-Duo plasmid to distinguish vacuolar from cytosolic *Salmonella* through the differential expression of mCherry and GFP fluorescent proteins (17). *Salmonella* strains were then cultured statically for 20 h at 37°C in LB supplemented with ampicillin 100 µg/mL. INT-407 and IPEC-J2 cells were infected for 1 h with ~1.5 × 10^5^ bacteria/mm^2^ at 37°C in 5% CO_2_ with breathable sealing membrane (Diversified Biotech BEM-1). A multiplicity of infection (MOI) based on the growth surface, rather than on the epithelial cell number, was applied to expose INT-407 and IPEC-J2 confluent monolayers to the same bacterial load, despite the different sizes of the two cell lines. After 1 h of infection, monolayers were treated with gentamicin 100 µg/mL for 1 h and gentamicin 10 µg/mL for the remaining time course of the infection. After 8 h of infection, monolayers were fixed with paraformaldehyde 4% (Sigma-Aldrich) for 20 min at room temperature and labeled with the HCS CellMask Blue cytoplasmic⁄nuclear stain following the manufacturer’s instructions (Invitrogen). Samples were imaged with a motorized Axio Observer Inverted Microscope with Colibri 5/7 light source (ZEISS) using a 20 ×/0.75 NA objective. The image analysis was performed with FIJI (NIH) (18) as described by Berni et al. (16). Briefly, epithelial cells were first segmented. Then, the percentage of cell area occupied by *Salmonella* expressing the mCherry constitutive reporter only or also the GFP cytosol-responsive reporter was measured. Cells were considered infected when the percentage of area occupied by *Salmonella* expressing the mCherry was ≥0.2%. The percentage of area occupied by *Salmonella* expressing the mCherry was used to calculate the mean vacuolar load. The percentage of the cell area occupied by GFP-expressing *Salmonella* was measured to quantify the cytosolic hyper-replication rate scored as the fraction of infected cells massively colonized by cytosolic *Salmonella* (considering only cells with the percentage of the occupied area ≥20% and ≥15% for human and swine cells respectively). The statistical analysis on the fraction of infected cells, vacuolar load, and hyper-replication rate was performed by one-way analysis of variance with Tukey’s correction for multiple comparisons on data from three biological replicates, each performed in triplicate.

## RESULTS

### Swine-adapted *Salmonella* serovars are subjected to extensive SPI-1 decay

**Fig 1:**
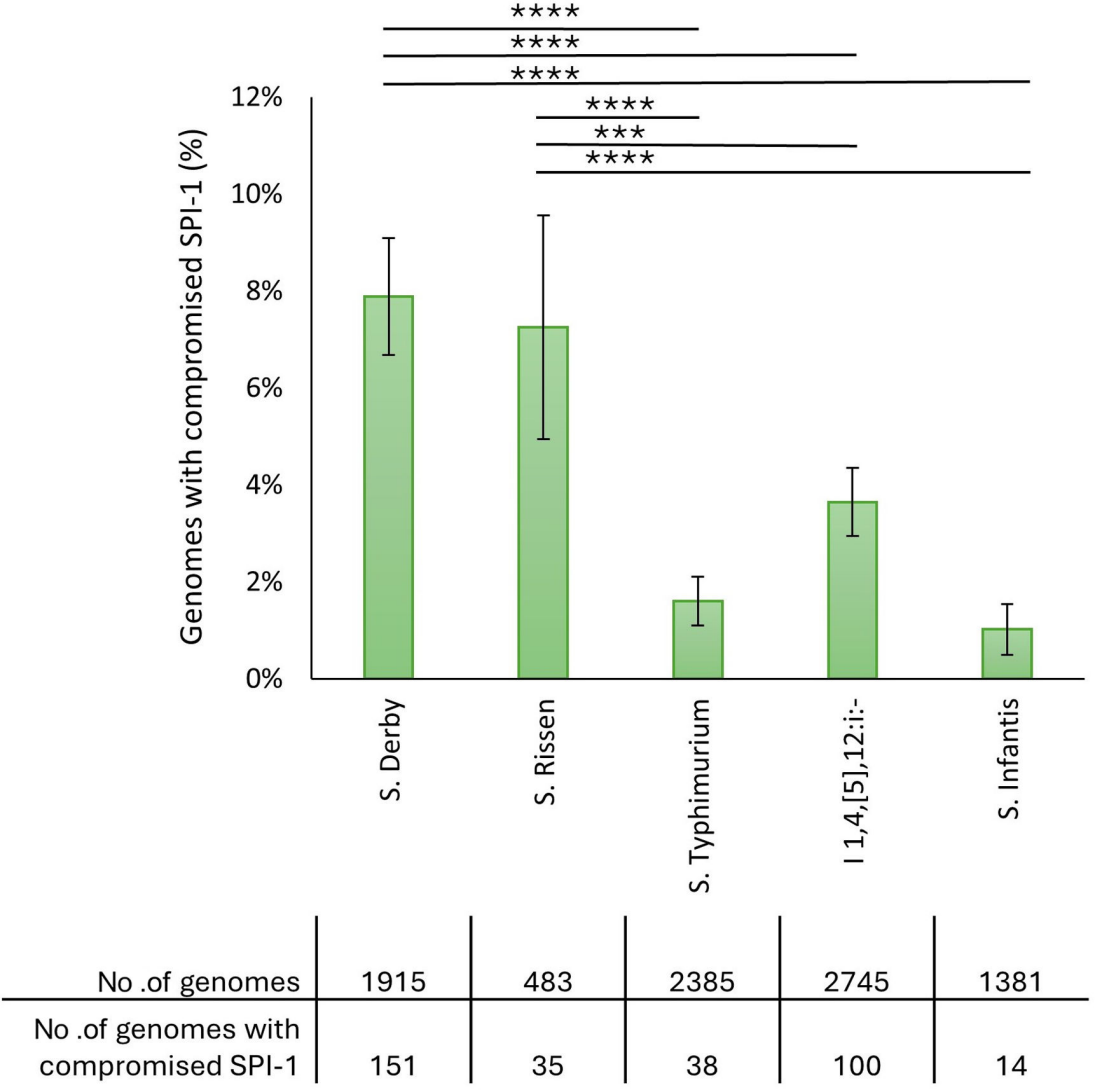
Analysis of genomes with compromised SPI-1 belonging to the serovars most isolated from swine. The chart shows the proportion of genomes with compromised SPI-1 for each serovar. Vertical bars indicate the 95% confidence intervals. Asterisks indicate statistically significant differences (****P < 0.0001, ***P < 0.001). The table reports the number of analyzed genomes and the number of genomes with missing or truncated SPI-1 genes for each serovar.

WgMLST data of 2,385 *S*. Typhimurium, 2,745 I 1,4,[5],12:i:-, 1,915 *S*. Derby, 1,378 *S*. Infantis, and 483 *S*. Rissen present in Enterobase and isolated from swine were examined to analyze the absence or truncation of SPI-1 genes in the serovars most frequently isolated from pigs. For each serovar, the proportion of genomes with at least one missing or truncated SPI-1 gene was calculated. The number of mutated genomes is reported in Fig. 1. *S.* Derby showed a significantly higher proportion of genomes with compromised SPI-1 compared with the generalist serovars *S*. Typhimurium (*P* < 0.0001), I 1,4,[5],12:i:- (*P* < 0.0001), and *S*. Infantis (*P* < 0.0001). A higher proportion of genomes with compromised SPI-1 was also observed in *S*. Rissen compared with *S*. Typhimurium (*P* < 0.0001), I 1,4,[5],12:i:- (*P* = 0.0007), and *S*. Infantis (*P* < 0.0001). The proportions of genomes with compromised SPI-1 were not significantly different between *S*. Derby and *S*. Rissen. These results suggest that swine-adapted serovars undergo converging evolution in their adaptation to pigs through the decay of SPI-1.

**Fig 2:**
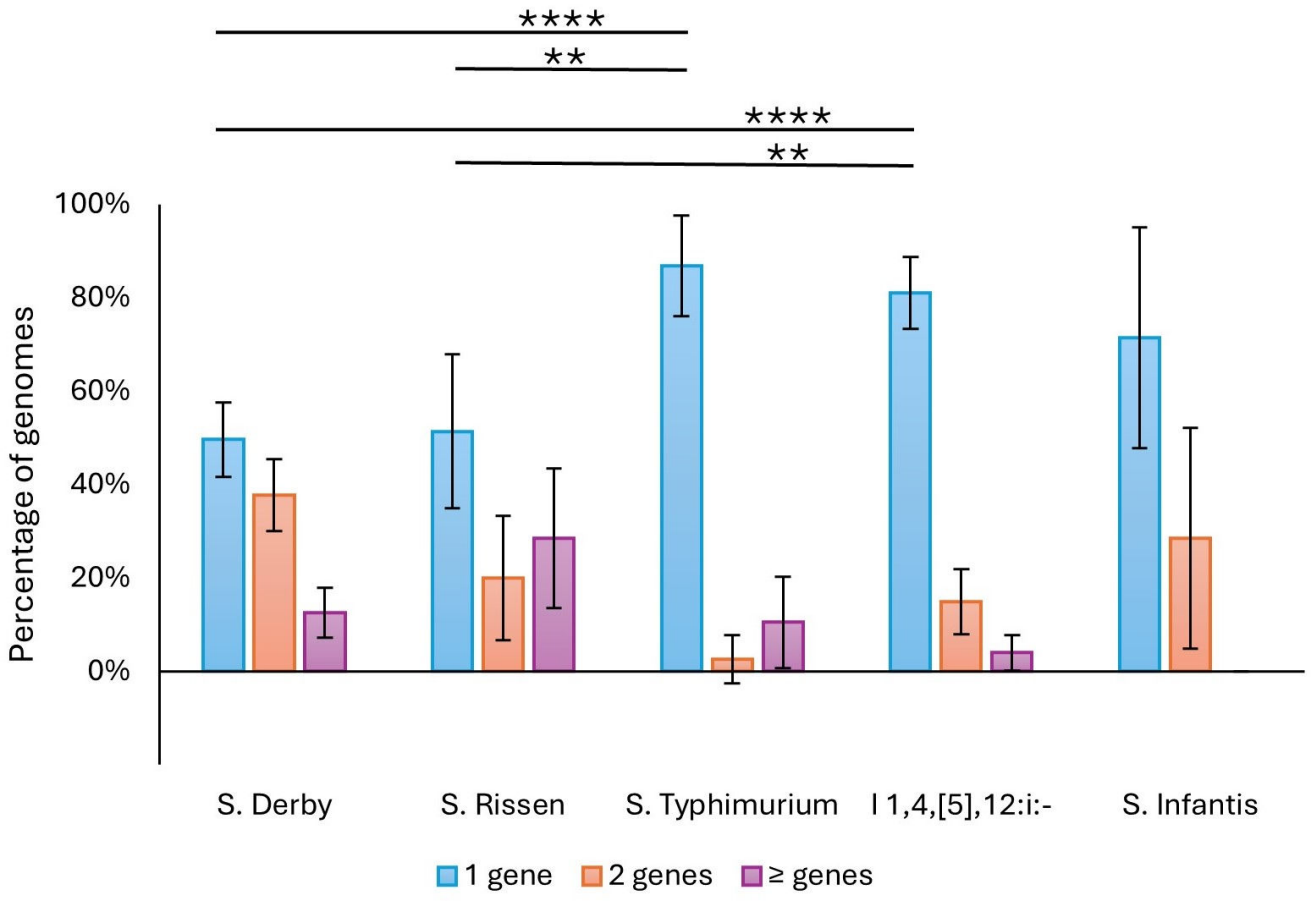
Percentage of genomes carrying 1, 2, or ≥3 missing/truncated SPI-1 genes among genomes with compromised SPI-1 for each serovar. Vertical bars indicate the 95% confidence intervals. Asterisks indicate statistically significant differences (****P < 0.0001, **P < 0.01).

Among the genomes with compromised SPI-1, the number of SPI-1 genes per genome that were missing/truncated was determined. The distribution of the number of missing/truncated genes per genome is shown in Fig. 2. Mutated *S*. Typhimurium has a higher proportion of genomes with only one such gene compared with *S*. Derby (*P* < 0.0001) and *S*. Rissen (*P* = 0.0072). This was the case also for I 1,4,[5],12:i:- compared with *S*. Derby (*P* < 0.0001) and *S*. Rissen (*P* = 0.006). No significant difference was observed for *S*. Infantis versus *S*. Derby and *S*. Rissen, likely because of the small number of *S*. Infantis genomes with compromised SPI-1. These results indicate that swine-adapted serovars undergo more extensive SPI-1 decay compared with generalist serovars.

**Fig 3:**
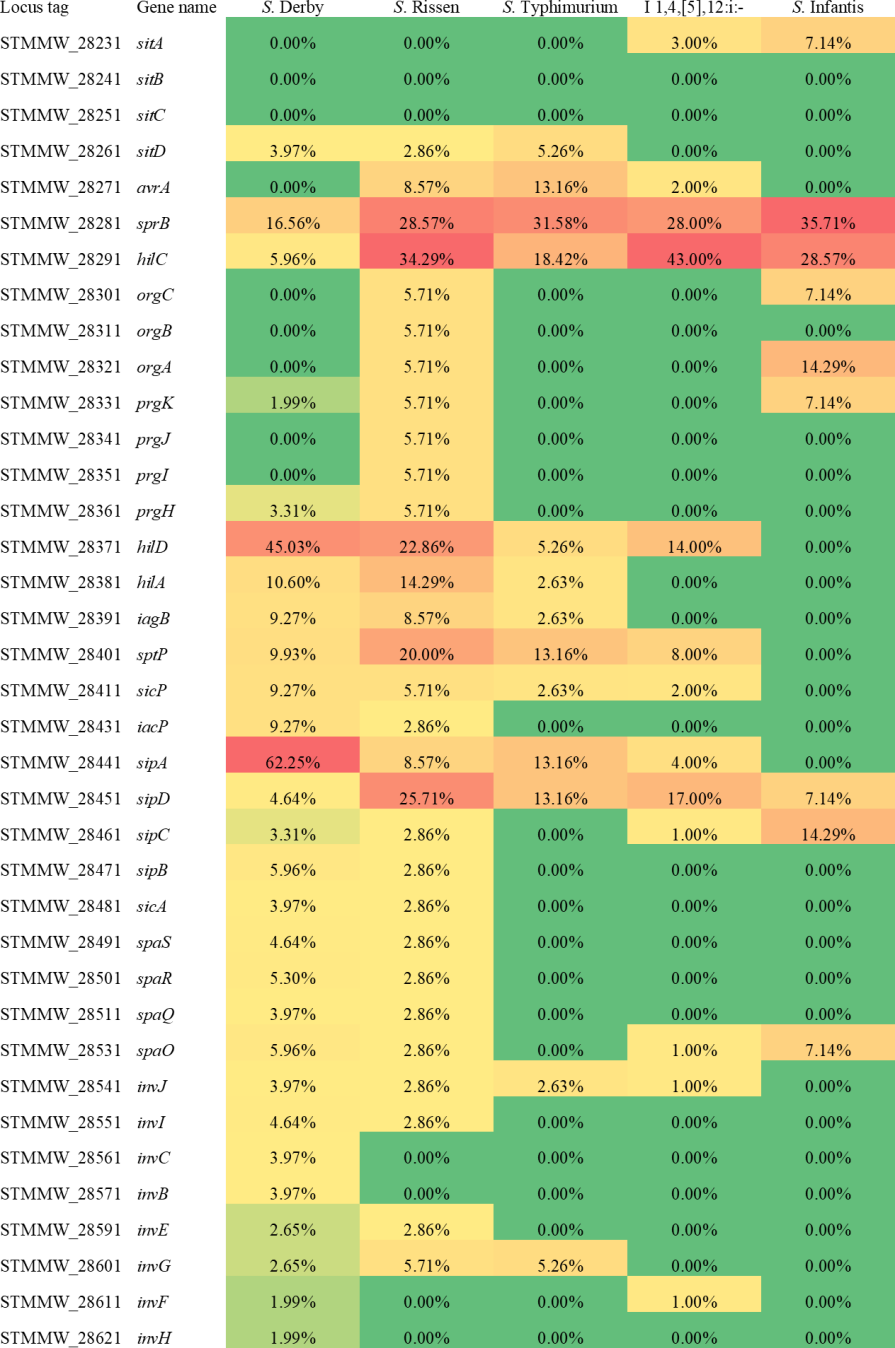
Percentage of genomes carrying lack/truncation in each SPI-1 gene among genomes with compromised SPI-1 for each serovar. For each gene, the cgMLST locus tag and the name are indicated. Red and green shadings represent higher and lower percentages, respectively.

We also investigated what genes in SPI-1 were more subject to loss/truncation (Fig. 3), and it can be observed that in *S*. Derby and *S*. Rissen, loss/truncation was detected in the majority of SPI-1 genes, whereas in *S*. Typhimurium, I 1,4,[5],12:i:-, and *S*. Infantis, several SPI-1 genes were never mutated. Interestingly, some genes were more frequently subject to loss/truncation in all analyzed serovars, that is, *sprB* and *hilC*, encoding for transcriptional regulators.

### Analysis of *S*. Derby and *S*. Rissen populations with missing or truncated SPI-1 genes

**Fig 4:**
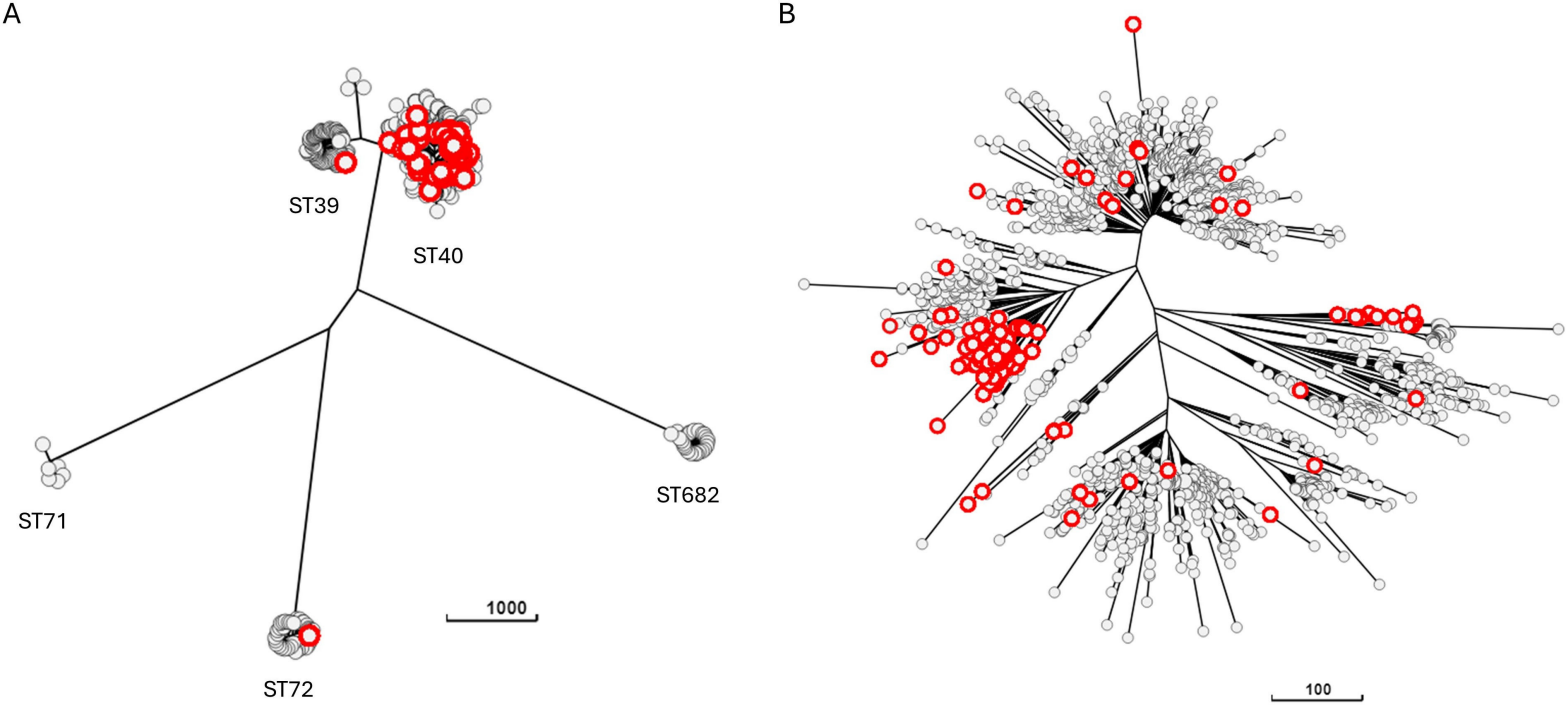
Neighbor-joining trees based on wgMLST allelic profiles and including all available *S.* Derby genomes (**A**) or only genomes belonging to ST40 (**B**). Nodes encircled in red represent the *S.* Derby genomes with missing/truncated SPI-1 genes.

We examined how genomes with truncation/mutation in SPI-1 genes were distributed in *S*. Derby population from swine. The neighbor-joining tree of *S*. Derby genomes, generated by using the Enterobase wgMLST scheme, showed five major lineages corresponding to five STs: ST39, ST40, ST71, ST72, and ST682 (Fig. 4A). All but two genomes with compromised SPI-1 (149/151) belong to ST40, the most diffused *S*. Derby sequence type in swine (5). A tree including only ST40 genomes was thus generated to analyze this sequence type in detail (Fig. 4B). The tree shows that genomes with compromised SPI-1 are present across the majority of lineages inside ST40. Most of the lineages containing mutants are composed of wild-type genomes together with a minority of mutants, whereas others are mostly made of mutated genomes. Two clusters were detected, which contain only genomes with missing/truncated SPI-1 genes. The largest of these clusters corresponds to one previously characterized carrying mutations in *sipA* (10).

**Fig 5:**
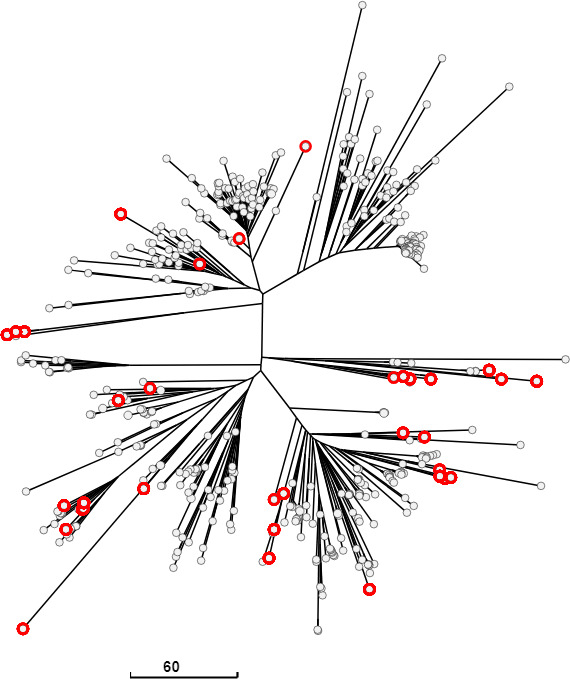
Neighbor-joining trees based on wgMLST allelic profiles and including all available *S.* Rissen genomes. Nodes encircled in red represent the *S.* Rissen genomes with missing/truncated SPI-1 genes.

The distribution of mutated genomes was investigated also in swine-associated *S*. Rissen (Fig. 5). Only genomes of ST469 were included in the analysis as almost all genomes (481 of 483) belonged to this sequence type. The neighbor-joining tree showed that genomes with missing/truncated SPI-1 genes were spread all over the tree branches, intermixed with the genomes with wild-type SPI-1.

### Effect of SPI-1 decay on *S*. Rissen behavior inside epithelial cells

It is commonly recognized that SPI-1 encodes the main mechanism used by *Salmonella* to colonize the intestinal epithelium. Indeed, the SPI-1 products are involved in each step of *Salmonella* pathogenesis inside intestinal epithelial cells, that is, invasion, survival in *Salmonella*-containing vacuole (SCV), replication inside SCV, and hyper-replication into the cytosol of cells(11).

**Fig 6:**
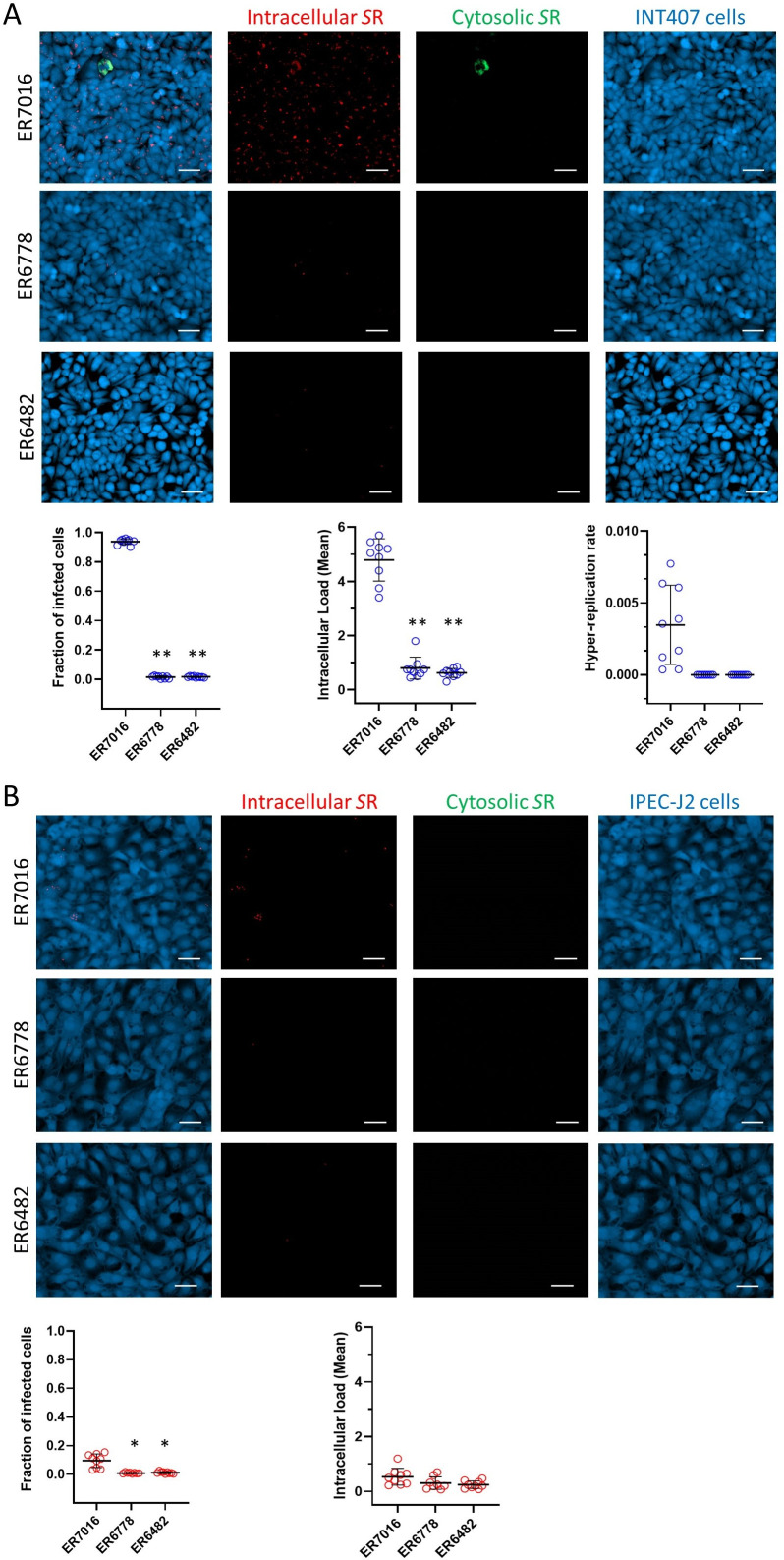
The results of INT-407 (**A**) and IPEC-J2 (**B**) cell infection assays. Microscopy images depict cells infected by *S.* Rissen (SR) ER7016, ER6778, and ER6482 strains carrying the pCHAR-Duo fluorescence reporter plasmid. Epithelial cells are shown in blue (HCS CellMask Blue), intracellular Salmonella in red (mCherry), and cytosolic hyper-replicating *Salmonella* in green (GFP). White scale bars are 50 µm. Diagrams report the fraction of infected cells, the mean vacuolar load, and the hyper-replication rate. Each dot represents a technical replicate. Horizontal bars indicate the mean of biological replicates. Vertical bars indicate standard deviation. Asterisks indicate a significant difference versus ER7016 (*P* < 0.001).

However, although the impact of SPI-1 on *S*. Derby virulence was previously studied, its role in *S*. Rissen was not previously evaluated. Therefore, cell culture infection assays were performed on human-derived INT-407 and swine-derived IPEC-J2 intestinal epithelial cells to confirm the role of SPI-1 also in this serovar. Invasion, vacuolar load, and cytosolic hyper-replication were quantified at a single-cell level by automated fluorescence microscopy. We tested one isolate carrying wild-type SPI-1 (ER7016) and two isolates that naturally lack the entire SPI-1 (ER6778 and ER6482). In human cells (Fig. 6A), ER7016 resulted highly virulent, infecting 94% of epithelial cells, compared with ER6778 and ER6482, which infected a mean of 1.5% and 1.7% of cells. Similarly, the vacuolar load, representative of the ability of *Salmonella* to survive and replicate inside SCV, resulted significantly higher for ER7016 compared with ER6778 and ER6482. We were able to quantify cytosolic hyper-replication only in cells infected by ER7016, whereas no hyper-replication was observed in cells infected by ER6778 and ER6482. These results pointed out that SPI-1 is essential in each step of human cell colonization also for *S*. Rissen.

Regarding swine cells (Fig. 6B), the percentage of cells infected by ER7016 (9.5%) resulted still higher than that of epithelial cells infected by ER6778 (0.9%) and ER6482 (1.2%) but considerably reduced compared with human cells (Fig. 6A). No difference in vacuolar load was detected among the tested isolates, with values for all tested isolates lower than in human cells. Furthermore, no cytosolic hyper-replication was detected for all *S*. Rissen strains.

### SPI-1 in the global population of poultry- and bovine-associated serovars

**Fig 7:**
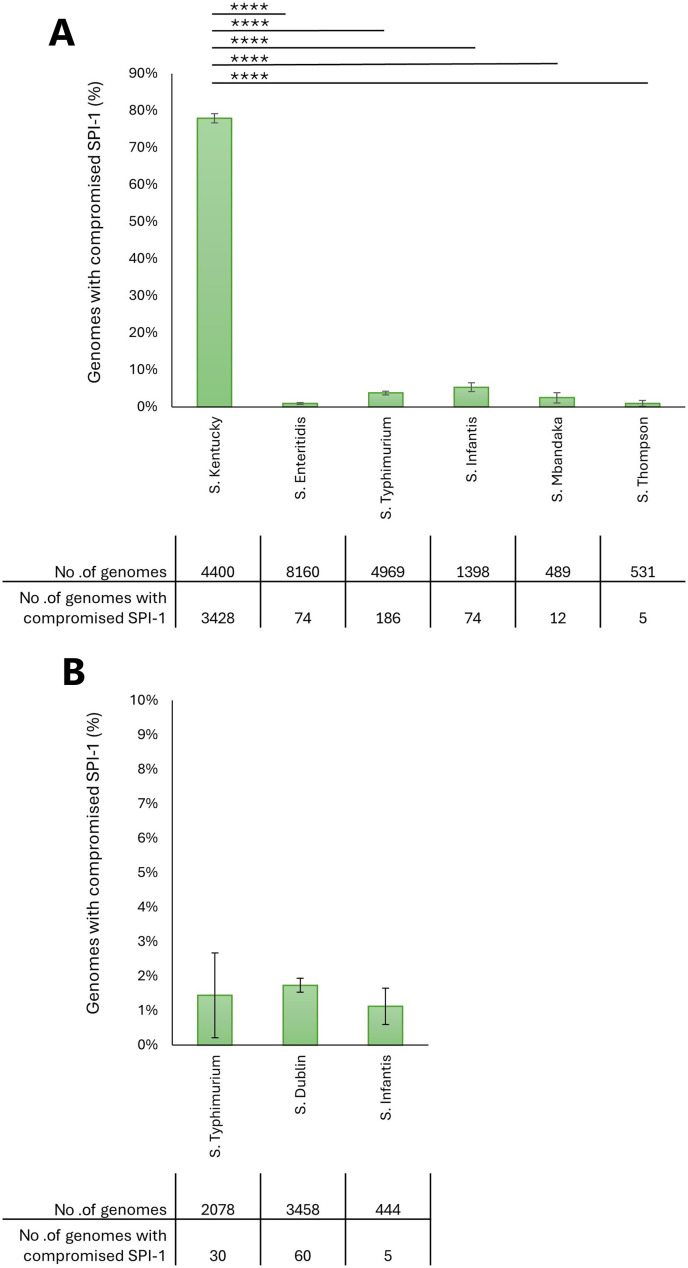
Analysis of genomes with compromised SPI-1 belonging to the serovars most isolated from poultry (**A**) and from cattle (**B**). The charts show the proportion of genomes with compromised SPI-1 for each serovar. Vertical bars indicate the 95% confidence intervals. Asterisks indicate statistically significant differences (****P < 0.0001). The tables report the number of analyzed genomes and the number of genomes with missing or truncated SPI-1 genes for each serovar.

The rate of deleterious mutations in SPI-1 was investigated also in serovars adapted to hosts other than swine. SPI-1 was analyzed in the serovars mostly isolated from poultry, namely *S*. Kentucky, a poultry-adapted serovar; *S*. Enteritidis, *S*. Typhimurium, *S*. Infantis, *S*. Mbandaka, and *S*. Thompson; and from cattle, *S*. Typhimurium, *S*. Dublin, and *S*. Infantis. Fig. 7A reports, for each poultry serovar, the number and proportion of genomes with at least one missing or truncated SPI-1 gene. It can be observed that the majority of *S*. Kentucky genomes (77.9%) carry deleterious mutations in SPI-1. The proportion of *S*. Kentucky genomes with compromised SPI-1 resulted significantly higher compared with the other serovars not adapted to poultry (*P* < 0.0001). The results about bovine serovars are reported in Fig. 7B. No bovine serovar was found with a significantly higher proportion of genomes carrying deleterious mutations in SPI-1 compared with the others.

### Analysis of *S*. Kentucky population with missing or truncated SPI-1 genes

**Fig 8:**
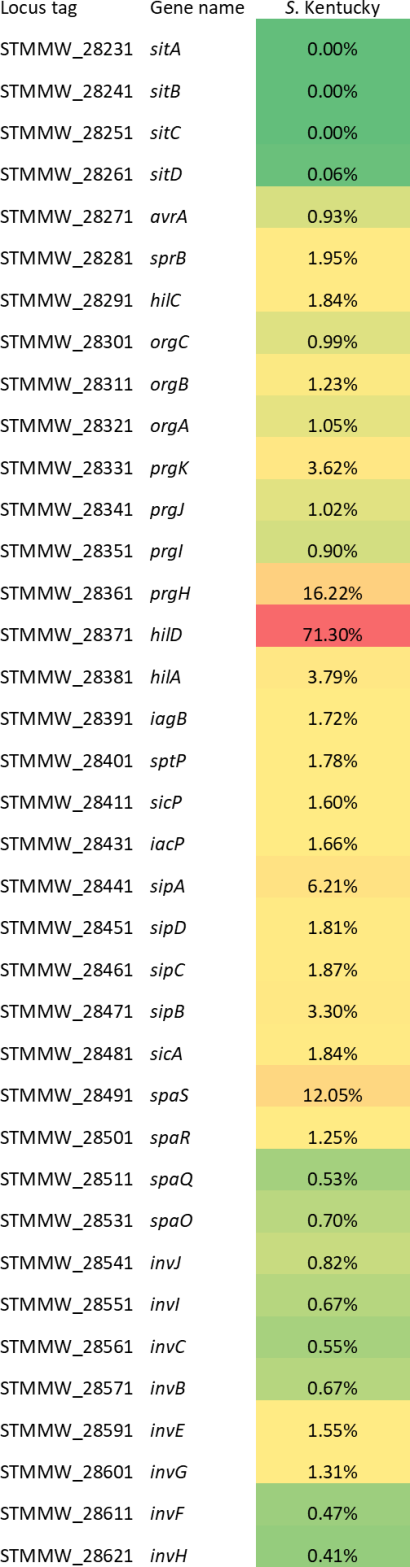
Percentage of genomes carrying lack/truncation in each SPI-1 gene among *S.* Kentucky genomes with compromised SPI-1. For each gene, the cgMLST locus tag and the name are indicated. Red and green shadings represent higher and lower percentages, respectively.

*S*. Kentucky genomes with compromised SPI-1 were analyzed to evaluate what SPI-1 genes were more subject to decay (Fig. 8). Loss/truncation was detected in most SPI-1 genes, in line with what was previously observed for *S*. Derby and *S*. Rissen. Notably, *hilD* resulted in the most mutated gene, with 71.3% of *S*. Kentucky genomes carrying a truncated allele. This gene encodes a master transcription regulator of SPI-1 (19).

**Fig 9:**
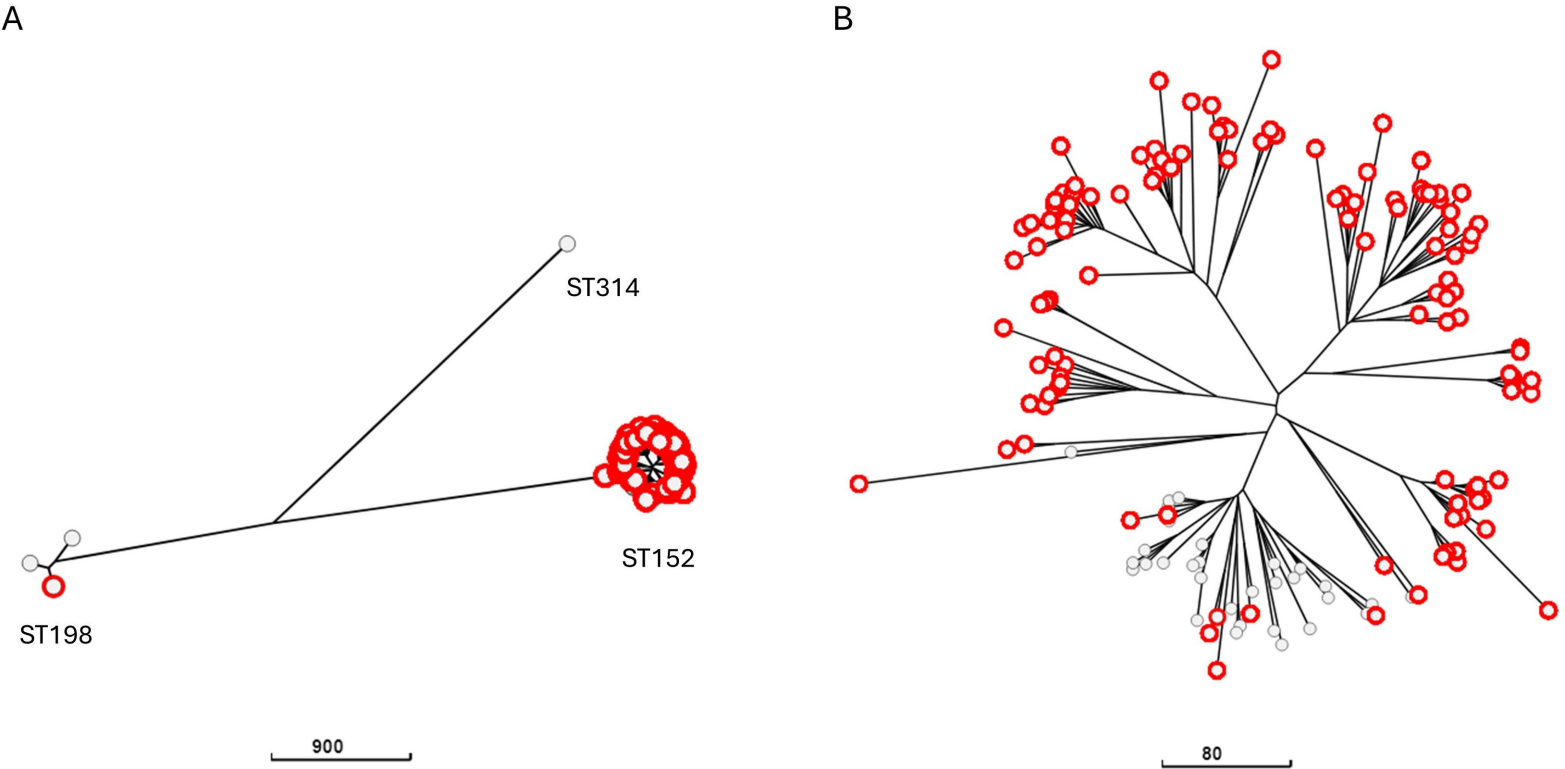
Neighbor-joining trees based on wgMLST allelic profiles and including a random selection of 149 *S.* Kentucky genomes (**A**) or only genomes belonging to ST152 included in the selection (**B**). Nodes encircled in red represent the *S.* Derby genomes with missing/truncated SPI-1 genes.

We next examined how genomes with truncation/mutation in SPI-1 genes were distributed in *S*. Kentucky population from poultry. The neighbor-joining tree of *S*. Kentucky genomes showed three major lineages corresponding to the following three STs: ST152, ST198, and ST314 (Fig. 9A). ST152 resulted in the most represented sequence type from poultry, as previously reported (7). A tree including only ST152 genomes was thus generated (Fig. 9B). All but one of the lineages of the tree resulted entirely composed of genomes with compromised SPI-1.

## DISCUSSION

Our results show that deleterious mutations in SPI-1 are significantly more accumulated in the population of swine-adapted (*S*. Derby and *S*. Rissen) than in generalist (*S*. Typhimurium, I 1,4,[5],12:i:-, and *S*. Infantis) serovars. In particular, not only the proportion of mutated genomes is higher in swine-adapted serovars but also the number of genes involved per genome is greater, indicating a more extensive SPI-1 decay compared with isolates from the same host species but belonging to generalist serovars.

The existence of SPI-1 mutated genomes in many branches of the phylogeny of *S*. Derby means that the occurrence and fixation of such mutations is a frequent event, resulting in the coexistence of mixed lineages with interspersed presence of mutated genomes as well as lineages composed exclusively by mutated genomes. Similarly, in *S*. Rissen, genomes with missing or truncated SPI-1 genes are spread all over the lineages and intermixed with the genomes with wild-type SPI-1.

In *S*. Derby, we already reported the role of mutated SPI-1 in reducing invasion and replication in intestinal epithelial cells. In our previous studies, we showed that two lineages, representing more than 30% of the *S*. Derby population from swine, carry deleterious mutations in the SPI-1 genes *hilC* and/or *sipA* associated with reduced virulence in intestinal epithelial cells (10). Our results confirm the same role of mutated SPI-1 in *S*. Rissen.

The evidence of the emergence of multiple genomic mutations of SPI-1 at population-scale, across different swine-adapted serovars, associated with an attenuated phenotype in intestinal cells defines a picture of converging evolution toward enhanced attenuation of these serovars.

*S*. Derby and *S*. Rissen are already known as generally attenuated serovars in both pigs and humans, and attenuation is not unexpected in association with adaptation as infection without disease preserves the host and consequently represents an advantage for the microorganism. In line with this, our study shows that a diffused process of further attenuation is currently ongoing, and this raises the question of why this extra-attenuation is taking place and what conditions are driving it. To look at this picture from a broader perspective, we also investigated the phenomenon in poultry, another reservoir for *Salmonella* in the food supply chain in addition to swine. Interestingly, we found that also the population of the poultry-adapted *S*. Kentucky is characterized by a very high proportion of genomes carrying SPI-1 mutations compared with not adapted serovars from poultry. Also, *S*. Kentucky is generally recognized as particularly attenuated for animals and humans (7, 8).

Conversely, this was not the case for another host-adapted serovar, *S*. Dublin, a bovine-associated *Salmonella*. For this specific host-pathogen combination, we are evidently in front of the type of adaptation that exists in association with increased virulence for the host as a strategy opposite to adaptation with attenuation. A similar type of host adaptation exists also in the case of *Salmonella* Choleraesuis with swine and *Salmonella* Typhi with humans. Not surprisingly, *S*. Dublin is highly pathogenic to humans too (20).

In regard to the question of why this extra-attenuation is taking place in many host-adapted serovars and what conditions are driving it, it can be observed that the modern animal production industry has led to the creation of large populations of very densely reared animals for several decades, and this condition could have changed the ecology of the serovars or lineages of *Salmonella* more closely associated with farmed animals. In particular, the need for high infectivity through the intestinal route could have diminished for these lineages in conditions where animal-to-animal contacts are extremely frequent and in an environment that is highly contaminated by animal feces. On the contrary, in these conditions, strains having reduced ability to infect the intestinal epithelium and being less virulent could be better tolerated by their hosts and ultimately more adapted. Accordingly, it was demonstrated by Kirchner and Roy (21) that, when the host and the pathogen have a relationship that is sufficiently specific for host-pathogen feedback to regulate pathogen trait frequencies, natural selection will favor reduced pathogen infectiousness.

The evolution through decay of SPI-1 as a possible adaptation process in serovars particularly associated with intensively farmed animals like pigs and poultry not only impacts the interaction of these species with *Salmonella* but also has repercussions on how the threat posed by *Salmonella* to humans is managed.

In this respect, the microbiological criteria for *Salmonella* in foods provided for in the legislation of many countries, for example, the EU Member States and the United States, ask for the absence of *Salmonella,* regardless of the serovar or genetic feature of the strains possibly present in the foodstuff (22). Based on our findings, evidence is accumulating on the substantial diversity of virulence among different lineages of some host-adapted serovars, with some of them showing increased attenuation. In light of this, a process to review the microbiological criteria for the management of *Salmonella* in animals and food could be considered taking into account that the risk posed to humans appears to be largely different in the presence of different lineages and so the measures to be taken to mitigate the risk.

## Data Availability

All genomic sequences used in this study are publicly available on Enterobase (https://enterobase.warwick.ac.uk) and on ENA (https://www.ebi.ac.uk/ena/browser/home) under Project number PRJEB75724.

## References

[1] European Food Safety Authority, European Centre for Disease Prevention and Control. 2022. The European union one health 2021 zoonoses report. EFSA J 20:e07666. doi:10.2903/j.efsa.2022.7666 36524203 PMC9745727

[2] Delahoy MJ, Shah HJ, Weller DL, Ray LC, Smith K, McGuire S, Trevejo RT, Scallan Walter E, Wymore K, Rissman T, McMillian M, Lathrop S, LaClair B, Boyle MM, Harris S, Zablotsky-Kufel J, Houck K, Devine CJ, Lau CE, Tauxe RV, Bruce BB, Griffin PM, Payne DC. 2023. Preliminary incidence and trends of infections caused by pathogens transmitted commonly through food — Foodborne Diseases Active Surveillance Network, 10 U.S. sites, 2022. MMWR Morb Mortal Wkly Rep 10:701–706. doi:10.15585/mmwr.mm7226a1 PMC10328488 37384552

[3] Stevens MP, Kingsley RA. 2021. Salmonella pathogenesis and host-adaptation in farmed animals. Curr Opin Microbiol 63:52–58. doi:10.1016/j.mib.2021.05.013 34175673

[4] Baumler A, Fang FC. 2013. Host specificity of bacterial pathogens. Cold Spring Harb Perspect Med 3:a010041. doi:10.1101/cshperspect.a010041 24296346 PMC3839602

[5] Sévellec Y, Vignaud ML, Granier SA, Lailler R, Feurer C, Le Hello S, Mistou MY, Cadel-Six S. 2018. Polyphyletic nature of Salmonella enterica serotype derby and lineage-specific host-association revealed by genome-wide analysis. Front Microbiol 9:891. doi:10.3389/fmicb.2018.00891 29867804 PMC5966662

[6] García-Fierro R, Montero I, Bances M, González-Hevia MÁ, Rodicio MR. 2016. Antimicrobial drug resistance and molecular typing of Salmonella enterica serovar Rissen from different sources. Microb Drug Resist 22:211–217. doi:10.1089/mdr.2015.0161 26295933

[7] Tate H, Hsu CH, Chen JC, Han J, Foley SL, Folster JP, Francois Watkins LK, Reynolds J, Tillman GE, Nyirabahizi E, Zhao S. 2022. Genomic diversity, antimicrobial resistance, and virulence gene profiles of Salmonella serovar Kentucky isolated from humans, food, and animal ceca content sources in the United States. Foodborne Pathog Dis 19:509–521. doi:10.1089/fpd.2022.0005 35960531

[8] Richards AK, Kue S, Norris CG, Shariat NW. 2023. Genomic and phenotypic characterization of Salmonella enterica serovar Kentucky. Microb Genom 9:001089. doi:10.1099/mgen.0.001089 37750759 PMC10569734

[9] Tambassi M, Berni M, Bracchi C, Scaltriti E, Morganti M, Bolzoni L, Tanner JR, Thilliez G, Kingsley RA, Pongolini S, Casadei G. 2020. Mutation of hilD in a Salmonella Derby lineage linked to swine adaptation and reduced risk to human health. Sci Rep 10:21539. doi:10.1038/s41598-020-78443-7 33299016 PMC7726570

[10] Berni M, Bolzoni L, Menozzi I, Dodi A, Bracchi C, Morganti M, Scaltriti E, Pongolini S, Tambassi M. 2023. Salmonella Derby adaptation to swine and simultaneous attenuation for humans go through decay of Salmonella Pathogenicity Island I. Microbiol Spectr 11:e0189923. doi:10.1128/spectrum.01899-23 37800927 PMC10715017

[11] Fattinger SA, Sellin ME, Hardt WD. 2021. Salmonella effector driven invasion of the gut epithelium: breaking in and setting the house on fire. Curr Opin Microbiol 64:9–18. doi:10.1016/j.mib.2021.08.007 34492596

[12] Fedorka-Cray PJ, Gray JT, Wray C. 2000. *Salmonella* in domestic animalsp 191–207. In Wray C, Wray A (ed), CAB International. UK.

[13] Boyen F, Pasmans F, Van Immerseel F, Morgan E, Adriaensen C, Hernalsteens J-P, Decostere A, Ducatelle R, Haesebrouck F. 2006. Salmonella Typhimurium SPI-1 genes promote intestinal but not tonsillar colonization in pigs. Microbes Infect 8:2899–2907. doi:10.1016/j.micinf.2006.09.008 17113332

[14] Zhou Z, Alikhan NF, Mohamed K, Achtman M. 2020. The EnteroBase user’s guide, with case studies on Salmonella transmissions, Yersinia pestis phylogeny, and Escherichia core genomic diversity. Genome Res 30:138–152. doi:10.1101/gr.251678.119 31809257 PMC6961584

[15] Zhou Z, Alikhan NF, Sergeant MJ, Luhmann N, Vaz C, Francisco AP, Carriço JA, Achtman M. 2018. GrapeTree: visualization of core genomic relationships among 100,000 bacterial pathogens. Genome Res 28:1395–1404. doi:10.1101/gr.232397.117 30049790 PMC6120633

[16] Berni M, Pongolini S, Tambassi M. 2022. Automated analysis of intracellular phenotypes of Salmonella using ImageJ. J Vis Exp 186. doi:10.3791/64263 36036620

[17] Cooper KG, Chong A, Starr T, Finn CE, Steele-Mortimer O. 2017. Predictable, inducible protein production in Salmonella for studying host-pathogen interactions. Front Cell Infect Microbiol 7:475. doi:10.3389/fcimb.2017.00475 29201859 PMC5696353

[18] Schindelin J, Arganda-Carreras I, Frise E, Kaynig V, Longair M, Pietzsch T, Preibisch S, Rueden C, Saalfeld S, Schmid B, Tinevez JY, White DJ, Hartenstein V, Eliceiri K, Tomancak P, Cardona A. 2012. Fiji: an open-source platform for biological-image analysis. Nat Methods 9:676–682. doi:10.1038/nmeth.2019 22743772 PMC3855844

[19] Saini S, Ellermeier JR, Slauch JM, Rao CV. 2010. The role of coupled positive feedback in the expression of the SPI1 type three secretion system in Salmonella. PLoS Pathog 6. doi:10.1371/annotation/df7e26bc-4c62-43b4-865f-a39274d98ab3 PMC2912647 20686667

[20] Kingsley RA, Bäumler AJ. 2000. Host adaptation and the emergence of infectious disease: the Salmonella paradigm. Mol Microbiol 36:1006–1014. doi:10.1046/j.1365-2958.2000.01907.x 10844686

[21] Kirchner JW, Roy BA. 2000. Evolutionary implications of host–pathogen specificity: the fitness consequences of host life history traits. Evol Ecol 14:665–692. doi:10.1023/A:1011647526731

[22] European Commission. 2005. Commission regulation (EC) No 2073/2005 of 15 November 2005 on microbiological criteria for foodstuffs. Off J Eur Union 338:1–26.

